# Unique microRNAs in lung adenocarcinoma groups according to major TKI sensitive EGFR mutation status

**DOI:** 10.1186/s13000-015-0339-4

**Published:** 2015-07-12

**Authors:** Min Gyoung Pak, Chang-Hun Lee, Woo-Jeong Lee, Dong-Hoon Shin, Mee-Sook Roh

**Affiliations:** Department of Pathology, Dong-A University Hospital, Busan, Republic of Korea; Department of Pathology and Medical Research Institute, Pusan National Univeristy Hospital, 1–10 Ami-dong, Seo-gu, Busan, 602-739 Republic of Korea; Department of Pathology, School of Medicine, Pusan National University, Yangsan, Republic of Korea; Department of Pathology, Dong-A University College of Medicine, Busan, Republic of Korea

**Keywords:** miRNA, miR-34c, miR-183, miR-210, NSCLC, Epidermal growth factor receptor

## Abstract

**Background:**

Lung cancer is the leading cause of cancer mortality, despite development of therapeutic strategies. Altered expression of microRNAs(miRNAs) in human malignancies have been well recognized as diagnostic and prognostic indicators, including lung cancer. This study aims to delineate the clinicopathologic significance of three unique miRNAs in adenocarcinoma according to major sensitive EGFR mutation status.

**Methods:**

One-hundred and three formalin-fixed paraffin-embedded (FFPE) tissues were collected from lung adenocarcinoma patients who underwent surgery and epidermal growth factor receptor *(EGFR)* mutation study. The samples were divided into three groups which include *EGFR* mutation in exons 19 and 21 and wild type. Some representative cases from each group were profiled using commercial miRNA microarray plates. Three significant miRNAs were selected and they were validated by quantitative real-time reverse transcription polymerase chain reaction (qRT-PCR), using collective cases of FFPE samples.

**Results:**

We identified three microRNAs (miR-34c, miR-183, and miR-210) which showed significantly altered expression in all groups of lung adenocarcinoma by microarray study. Compared to normal control lung tissue, down-regulation of miR-34c and up-regulation of miR-183 and miR-210 were identified in caner groups (*p* < 0.05 for each). We validated the expression of three miRNAs by qRT-PCR. Expression levels of miR-34c, miR-183, and miR-210 were significantly different between normal control group and cancer groups (*p* = 0.034, <0.000, and 0.036, respectively). Moreover, expression level of miR-183 was significantly higher in *EGFR* mutation groups than wild type group (*p* = 0.028). Higher expression levels of three miRNAs were positively related to poor tumor differentiation. Increased expression of miR-183 was positively associated with lymphovascular invasion (*p* = 0.037). Aberrant expression of miR-210 was independently associated with T stage (*p* = 0.019), and TNM stage (*p* = 0.007). However, there was noted a limited statistical significance. In *EGFR* exon 19 mutation group, miR-34c high expression group showed poor overall survival than low expression one by univariate Kaplan-Meier method. (*p* = 0.035).

**Conclusions:**

Here, we show that miR-34c may act as a potential tumor suppressor gene and miR-183 and miR-210 have a potential oncogenic role in pulmonary adenocarcinoma. This study also suggests different miRNA expression between *EGFR* mutation group and wild type group. Consequently, further studies of the biology of miRNAs may lead to diagnostic and prognostic biomarkers in pulmonary adenocarcinoma.

## Background

Lung cancer is the major leading cause of cancer mortality [1], and non-small cell lung cancer (NSCLC) occupies about 80 % of lung cancer. Despite development of various therapeutic strategies, complete surgical resection is still the treatment of choice of NSCLC [2]. More personalized and targeted treatments have been being developed, even now. In sync with those trials, altered expression of microRNAs (miRNAs) in human malignancies is recently identified as diagnostic and prognostic indicators, including lung cancer [3].

miRNAs are small non-coding RNA gene products about 21–25 nucleotides long [4], that negatively regulate gene expression. Single miRNA regulates hundreds of downstream signaling pathways [5], and it can influence several cellular key processes, including cellular development, differentiation, proliferation, cell death, and metabolism [6]. As predictably, miRNAs play a pivotal role as oncogenes or tumor suppressors in many kinds of cancers [3]. Zhang et al. suggest that several miRNAs are directly involved in lung, breast, brain, liver, colon cancer, and leukemia [3], specific miRNAs can be useful diagnostic and prognostic biomarkers [6]. In lung cancer, miR-99b and miR-102 showed higher expression levels in adenocarcinoma than squamous cell carcinoma, adenocarcinoma patients with high miR-155 and low let-7a-2 expression were related with poor survival [7]. Increased expression of miR-708 in never smoker lung adenocarcinoma was associated with poor overall survival [8].

NSCLC with somatic mutations in the tyrosine kinase domain of the epidermal growth factor receptor (*EGFR*) gene are associated with good responsiveness to tyrosine kinase inhibitors (TKIs), gefitinib and erlotinib [9]. Two major types of *EGFR* mutations, exon 19 deletions and exon 21 L858R substitutions, are the most frequent mutations, representing 85 % to 90 % of *EGFR* mutations reported [10]. But, vast majority of NSCLCs initially respond to *EGFR* inhibitors become resistant to these drugs [11]. miRNAs emerge as an independent predictor of the response to the drug. Shen et al. identified that patients with reduced miR-21 after receiving adjuvant gefitinib therapy showed a significant improvement in overall survival [12].

But, there are still limited studies regarding roles of miRNAs in NSCLC according to specific tumor type or genetic status.

This study aims to delineate the clinicopathologic significance of unique miRNAs in adenocarcinomas classified according to major tyrosine-kinase inhibitor sensitive *EGFR* mutation status. The cases were divided into three groups which include *EGFR* mutation in exons 19 and 21 and wild type. Several representative cases from each group were profiled using commercial miRNA microarray plates. Clinicopathologic significance of unique miRNAs were validated statistically by quantitative real-time reverse transcription polymerase chain reaction (qRT-PCR) using a large number of formalin-fixed paraffin-embedded (FFPE) specimens of NSCLC.

## Methods

### Patients and tissue samples

**Fig. 1:**
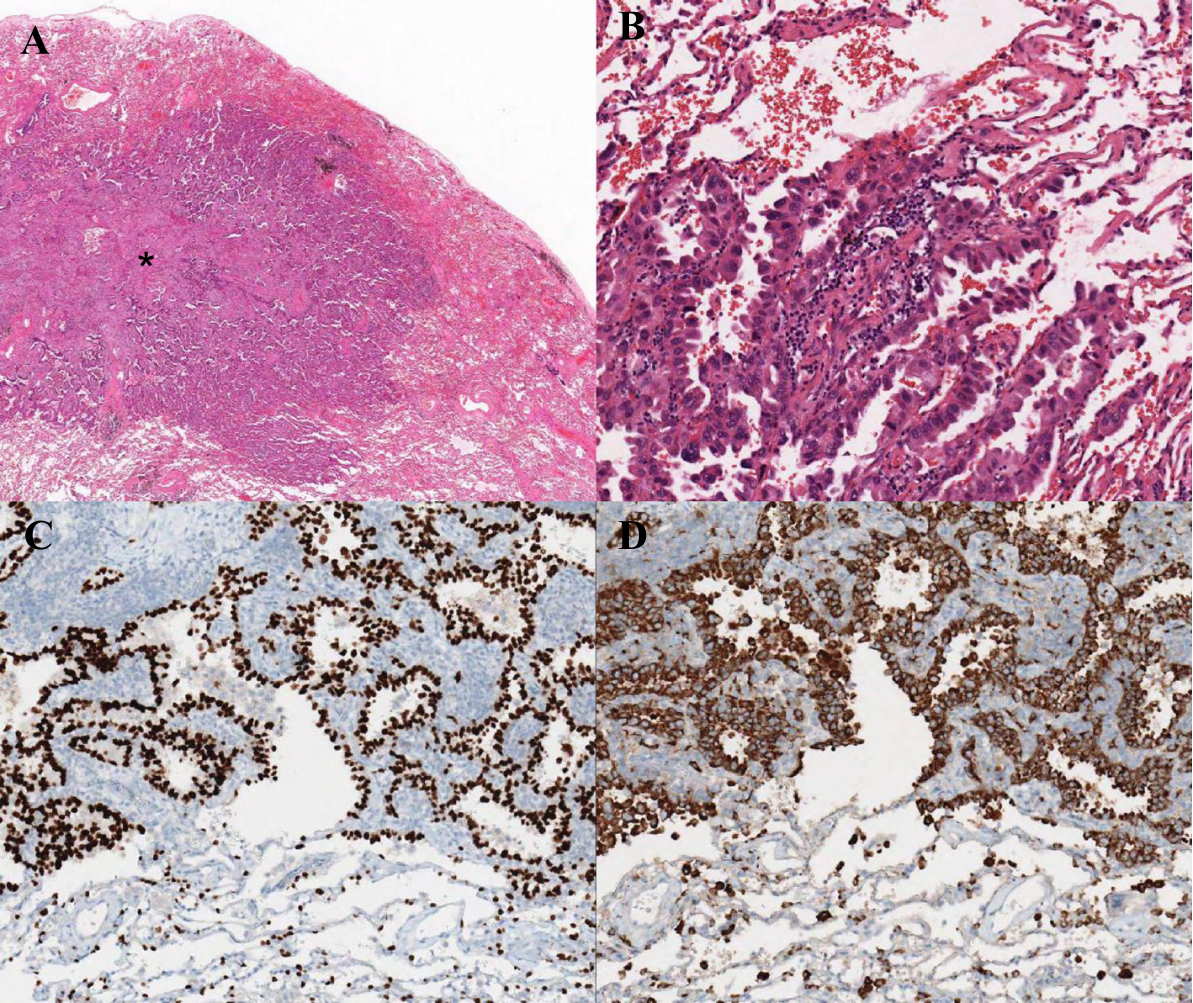
Histologic findings of primary lung adenocarcinoma. **a** Low magnification of an adenocarcinoma that has a central scar (*) (H&E). **b** High magnification of the same case (H&E): cuboidal to columnar shaped cells grow in a lepidic pattern along with alveolar wall. **c** TTF-1 imunohistochemical stain: diffuse strong nuclear positivity of tumor cells. **d** Napsin A imunohistochemical stain: diffuse strong granular cytoplasmic positivity of tumor cells

One-hundred and three FFPE tissues were collected from primary lung adenocarcinoma patients who underwent surgery and epidermal growth factor receptor *(EGFR)* mutation study from January 2008 to December 2012 at Pusan National University Hospital (PNUH), Busan, South Korea. Eligible samples were obtained from primary lung adenocarcinoma without preoperative chemotherapy or radiotherapy history. Normal control lung tissues were harvested from areas of more than 5 cm apart from main tumor mass, and they were histologically confirmed normal lung tissue without tumor infiltration, pulmonary infection, or inflammation. All patients had a preoperative chest computed tomographic (CT) scan, whole body bone scan and positron emission tomography with fluorodeoxyglucose (FDG-PET) in order to rule out the possibility of metastatic lung tumor or co-existing malignancy. Besides, mixed histology and patients with co-existing malignancy were excluded. Further immunohistochemical stains for thyroid transcription factor-1 (1:2, TTF-1; SP141, Ventana, Tucson, AZ, USA) and napsin A (1:300, clone IP 64, Leica Biosystems, Newcastle Upon Tyne, England), known as reliable markers for adenocarcinoma of lung origin [13], were performed using an automated immunostainer (Benchmark XT, Ventana, Tucson, AZ, USA) (Fig. 1). Along pyrosequencing result about *EGFR* mutational status (Green Cross Corp., Seoul, Korea), the samples were subclassified into *EGFR* exon 19 mutation group, *EGFR* exon 21 mutation group, and wild type group. Details of clinicopathologic data are illustrated on Table 1. This study was approved by the institutional review board of PNUH (PNUH IRB approval No. E-2014016).

**Table 1:** Clinicopathologic factors of lung adenocarcinomas in this study

Parameter	Exon 19 mutation	Exon 21 mutation	Wild type
Age at diagnosis	62.65	64.90	69.75
Sex			
Male	15	17	6
Female	23	23	6
T stage			
1a	14	20	7
1b	8	12	2
2a	14	2	3
2b	1	1	0
3	1	5	0
4	0	0	0
N stage			
0	31	35	10
1	3	3	2
2	4	2	0
M stage			
0	38	38	11
1	0	2	1
Stage			
IA	19	20	7
IB	10	12	2
IIA	5	2	3
IIB	0	1	0
IIIA	4	5	0
IIIB	0	0	0
Pleural invasion			
PL0	33	35	10
PL1	4	4	2
PL2	0	0	0
PL3	1	1	0
LVI^a^			
No	30	35	10
Yes	8	5	2
Growth pattern			
Lepidic	19	25	5
Acinar, papillary	15	12	7
Solid, Micropapillary	4	3	0
Micropapillary growth pattern			
No	32	39	11
Yes	6	1	1
Smoking			
Never	31	35	10
Ever	3	3	2
Current	4	2	0
Smoking (PY^b^)	14.8	9.6	23.9
Follow-up (months)	27.1	20.1	21.5

^a^LVI (lymphovascular invasion)

^b^PY(pack year)

### RNA isolation

Total RNAs were isolated from 103 primary lung adenocarcinoma (13 for microarray and 90 for RT-qPCR analysis) and 12 normal control lung tissues (one for microarray and 11 for RT-qPCR analysis), Unstained FFPE tissues from of tumors and control samples were sectioned to 10-μm thickness and 2 to 4 tissue sections were placed into a 1.5-mL tube. Xylene (1 mL) was added for deparaffinization and vortexed vigorously at room temperature for 3 minutes. After the ethanol series to remove xylenes, RNA was extracted with the miRNeasy FFPE kit (Qiagen®, Hilden, Germany). Briefly, lysis buffer allows tissue lysis with protinase K digestion in 15 minutes, After lysis, tissue samples were incubated at 80 °C for 15 minutes, and treated with DNase booster buffer. Concentrated RNA is purified using a RNeasy MinElute spin column, and quantified with a NanoDrop spectrophotometer (ThermoFisher Scientific®, Waltham, MA, USA). Abundance and integrity of 18S and 28S ribosomal bands were assessed via the Agilent 2100 Bioanalyzer (Agilent Technologies®, Palo Alto, CA, USA). After extraction, all RNA samples were stored at −80 °C until used.

### MicroRNA microarray and data analysis

This study for miRNA screening was performed using miScript miRNA PCR array Human Cancer Pathway Finder (Cat.No.MIHS-102Z, Qiagen®, Hilden, Germany) that contained selected human cancer specific miRNAs. cDNA was synthesized from 500 ng of total RNA using the miScript II Reverse Transcription Kit (Cat.No.218161), and miRNAs were isolated using the commercially available kits, miRNeasy FFPE Kit (Cat.No.73504, Qiagen®, Hilden, Germany) according to manufacturer’s instructions. Quantification of miRNAs was performed using the miScript SYBR Green PCR kit (Qiagen®, Hilden, Germany) according to manufacturer’s recommendations on a 7500 Real-Time PCR System (Applied Biosystems®, Foster City, CA, USA). We analyzed data by the miScript miRNA PCR Array Data Analysis Program of Qiagen®.

### RT-qPCR of miRNA derived from primary lung adenocarcinomas and normal control lung tissues

Three miRNAs, miR-34c, miR-183, and miR-210, which with showed statistical differences in their expression among three groups, were selected. They were quantified and validated with a larger sample group than miRNA microarray sample group by quantitative real-time reverse transcription polymerase chain reaction (qRT-PCR) from 90 primary lung adenocarcinoma and 11 normal control lung tissues. The reverse transcription (RT) reaction was carried out with a TaqMan MicroRNA Reverse Transcription Kit (Applied Biosystems®, Foster City, CA, USA) according to the instruction of the protocol. One to ten nanograms of total RNA per 15 μL RT reaction were processed at 16 °C for 30 minutes, 42 °C for 30 minutes, and 85 °C for 5 minutes. Following the RT, quantitative real-time reverse transcription polymerase chain reaction was carried out with a TaqMan® Universal PCR Maser Mix II (no UNG; uracil-N glycosylase, Applied Biosystems®, Foster City, CA, USA) according to manufacturer’s instructions with the 7500 Real-Time PCR System (Applied Biosystems®, Foster City, CA, USA) at 95 °C for 10 minutes, followed by 40 cycles of 95 °C for 15 seconds and 60 °C for one minute.

Each sample was examined in triplicate and analyzed by the comparative threshold cycle (Ct) method. The Ct values were calculated with Sequence Detection Software (SDS version 2.0.1, Applied Biosystems®, Foster City, CA, USA). The average expression levels of miRNAs were normalized with RNU48 (SNORD48) as an endogenous control gene, using the 2^-ΔΔCt^ method [14]. The mean Ct value in three miRNAs was calculated, excluding outliers (i.e., replicates with a Ct differing by > one cycle from the median). If Ct_RNU48_, _ave_ was not within 20 and 32 cycles, the analysis was repeated. All TaqMan probes were purchased form Applied Biosystems; has-miR-34c (Assay ID 000428), has-miR-183 (ID 002270), has-miR-210 (ID 000512).

#### Statistical analyses

Statistical analyses were conducted using the statistical software SPSS 18.0 for Windows (SPSS Inc., Chicago, IL, USA). The chi-square test was used to assess miR-34c, miR-183, miR-210 expression with respect to clinicopathological parameters. The Mann–Whitney test (2 groups) and Kruskal-Wallis test (n groups) were used to compare the differences between groups. The survival curves of the patients were determined using the Kaplan-Meier method and Cox regression, and the log-rank test was used for statistical evaluations. A probability (*P*)-value less than 0.05 was considered statistically significant.

## Results

### MicroRNA microarray analysis

Microarray analysis was used to detect some physiologically relevant miRNAs in lung adenocarcinoma. MicroRNA arrays (miScript miRNA PCR Array Human Cancer Pathway Finder, Qiagen®) that contained 84 human miRNA probes were performed from thirteen lung adenocarcinoma, including five *EGFR* exon 19 mutation patients, five *EGFR* exon 21 mutation ones, three wild type ones, and one normal control lung tissue. We identified three microRNAs (miR-34c, miR-183, and miR-210) which showed significant altered expression in all groups of lung adenocarcinoma by microarray study. Compared to normal control lung tissue, down-regulation of miR-34c and up-regulation of miR-183 and miR-210 were identified in caner groups (*p* < 0.05 for each).

### MicroRNA expression and *EGFR* mutation status

**Fig. 2:**
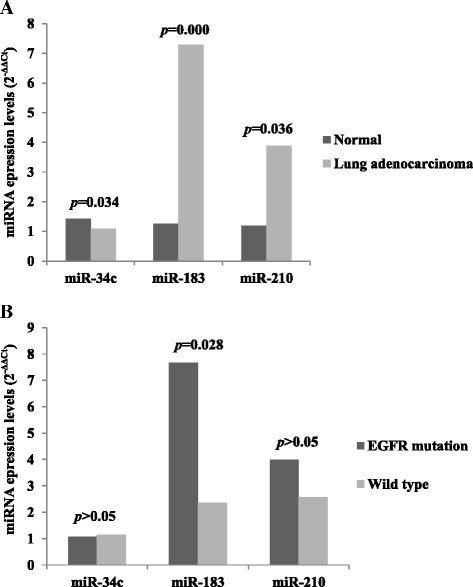
**a**. miRNAs expression levels between normal tissue and lung adenocarcinomas of the lung. **b** miRNAs expression levels between *EGFR* mutation and wild type adenocarcinomas of the lung

We validated the expression of three miRNAs in ninety lung cancers, consisting of thirty-eight *EGFR* exon 19 mutation patients, forty *EGFR* exon 21 mutation ones, twelve wild type ones, and eleven normal control lung tissue, by qRT-PCR. Expression levels of miR-34c, miR-183, and miR-210 were significantly different between normal control group and cancer groups (*p* = 0.034, <0.000, and 0.036, respectively; Fig. 2a). Down-regulation of miR-34c and up-regulation of miR-183 and miR-210 were identified in cancer groups. Expression levels of miR-183 were significantly higher in *EGFR* mutation groups than wild type group (*p* = 0.028; Fig. 2b). But miR-34c expression levels were similar between two groups, and miR-210 showed not significant but higher expression tendency in *EGFR* mutation groups than wild type. There was no statistical difference in the expression of three miRNAs between *EGFR* exon 19 and exon 21 mutation groups.

### MicroRNA expression and clinicopathologic parameters

We analyzed the correlation between miRNAs and clinicopathologic parameters, including age, sex, smoking history, TNM stage, tumor growth pattern, pleural invasion, and lymphovascular invasion to evaluate the potential role of these miRNAs in demographic pattern and progression of lung adenocarcinoma. In this paper, adenocarcinomas with predominantly lepidic growth pattern were titled as well-differentiated tumor, with acinar or papillary growth as moderately-differentiated tumor, and with solid or micropapillary predominance as poorly-differentiated tumor, in short. Tumor differentiation was significantly associated with miR-34c, miR-183, and miR-210 expression levels (*p* = 0.037, 0.029, and 0.003, respectively). Higher expression levels of three miRNAs were positively related to poor tumor differentiation. Increased expression of miR-183 was also positively associated with lymphovascular invasion (*p* = 0.037). Increased miR-210 expression was found to be significantly associated with T stage (*p* = 0.019), TNM stage (*p* = 0.007), and showed a higher tendency toward pleural invasion (*p* = 0.094). Additionally, heavy smokers have a tendency to show high expression level of miR-210. But, no significant correlation was observed between the expression levels of three miRNAs and age, sex.

### MicroRNA expression and overall survival

**Fig. 3:**
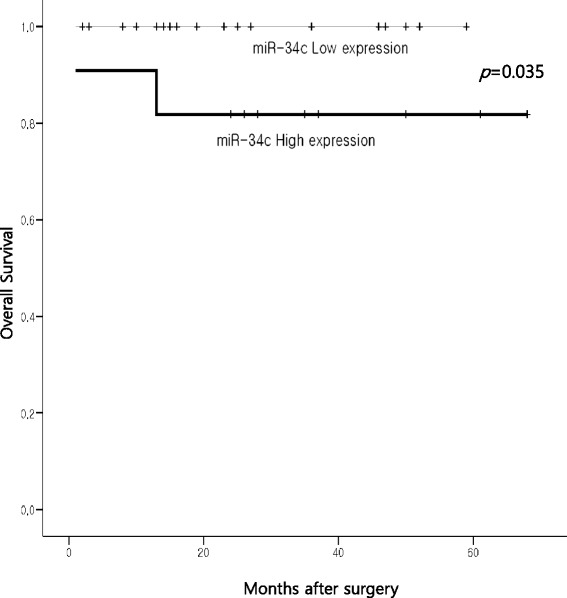
Comparison of overall survivals according to miR-34c expression level in lung adenocarcinomas showing *EGFR *exon 19 mutation

Overall survival time was calculated from the date of the initial surgical operation to last visit or death. Follow-up duration is 1 to 68 months (average 23.3 months, 27.1 months for *EGFR* exon 19 mutation patients, 20.1 months for *EGFR* exon 21 mutation ones, 21.5 months for wild type ones). To investigate the potential biologic roles of three miRNAs, we further subclassified lung adenocarcinomas into “high” and “low” groups in the expression of miR-34c, miR-183, miR-210, based on the mean of expression after normalization [8], and performed survival analysis by univariate Kaplan-Meier method. However, there was noted a limited statistical significance. In *EGFR* exon 19 mutation group, miR-34c high expression group showed poor overall survival than low expression one (*p* = 0.035, Fig. 3). In other two miRNAs, however, there is no significant relationship between overall survival and expression levels.

## Discussion

Despite the development of early detection and surgical techniques, lung cancer is still the leading cause of cancer-related death. Recent advances in targeted therapy have provided us with new treatment options. The oral tyrosine kinase inhibitors that target the *EGFR*, gefitinib and erlotinib, have been opened a new chapter in the treatment of NSCLC [15]. Lung cancer patients with *EGFR* mutation (ie, exon 19 deletions or exon 21 L858R point mutations) achieve a substantially increased benefit from treatment with *EGFR* tyrosine kinase inhibitor, compared with standard chemotherapy [16]. Despite the dramatic responses to *EGFR* inhibitor, many patients may have an ultimate relapse [17]. In addition, staging systems of lung cancer may have reached their limit of usefulness for predicting outcomes [5]. The fact is we desperately need more accurate and effective treatment target, subclassification of lung cancer, and additional molecular biomarkers, such as miRNAs, would be useful in determining an individualized treatment plan for lung cancer and subclassification of lung cancer according to molecular biomarkers. Herein, we identified that miR-34c, miR-183, and miR-210 showed significantly altered expression in some lung adenocarcinomas.

The miR-34 family is composed of three miRNAs (miR-34a, miR-34b, and miR-34c) that are direct transcriptional targets of p53, so their expression is induced by p53 in response to DNA damage and oncogenic stress [18, 19]. The transcriptional start site of miR-34c is adjacent to a predicted p53 binding site, transcriptional activation of miR-34c by p53 induces post-transcriptional gene silencing of cyclin-dependent kinase(CDK)-4, CDK-6, cyclinE2, and Bcl2 [19]. miR-34c expression was repressed in lung cancer cell lines [20] and other human malignancies, including colorectal, breast, melanoma, head and neck, and prostate cancers [21]. We found that miR34c showed significant down expression in lung adenocarcinoma tissue than normal lung, indicating tumor suppressor role of miR-34c. Paradoxically, however, there was a positive correlation between miR-34c expression level and poor tumor differentiation. Despite a limited statistical data, high expression group of miR-34c also showed poor overall survival than low expression one, especially in *EGFR* exon 19 mutation lung cancer patients. Thus, we consider that miR-34c may have opposite role in a particular condition. In spite of limited results, we assume that miR-34c acts like-a tumor suppressor gene in tumorigenesis, but in a particular condition, it may contribute tumor progression. Catuogno et al. demonstrated that miR-34c-5p may confer resistance to caspase-8-induced apoptosis by silencing of Bmf (Bcl-2-modifying factor), and miR-34c-5p controls cell proliferation and apoptosis by acting on p53 and c-myc [22]. It is an intriguing question that which mechanisms determine miR-34c to have opposite roles on cell survival or apoptosis. There is no definite evidence, but the action of miR-34c may depend on biologic circumstance. We presume that miR-34c acts differently depending on p53 status. There are some data that central tumor suppressor, p53, modulates several miRNAs expression, including miR-16-1, miR-143 and miR-145 [18, 23, 24]. Furthermore, p53 overexpression of lung cancer is known as a poor prognostic indicator [25, 26]. Ohsaki et al. suggested EGFR expression correlates with poor prognosis in NSCLC patients with p53 overexpression [27]. In this context, it can be a reasonable excuse that high expression group of miR-34c shows poor overall survival in *EGFR* exon 19 mutation patients.

There were no proven correlations between miR-34c expression and tumor stage, pleural involvement, lymphovascular invasion, smoking history in this study. There should be a rise of research that reveals correlation between miRNAs and many clinicopathologic parameters. miR-34c expression levels were similar between *EGFR* mutation group and wild type group. It seems that there is no relationship between miR-34c and *EGFR* mutation provisionally, but further studies are needed from now on.

miR-183 belongs to one of the unique miRNAs in lung adenocarcinoma [28], and it has a potential oncogenic role in conjuction with two tumor suppressor genes, *EGR1* and *PTEN* [29]. In patients with lung cancer, overexpression of miR-183 is associated with poor overall survival [30]. Xu et al. suggested that up-regulation of miR-183 in female lung adenocarcinoma was found to be associated with lymph node metastasis, advanced clinical stage, *EGFR* mutation, poor overall survival and progression-free survival [31]. We revealed that miR-183 showed significant higher expression in lung adenocarcinoma tissue than normal lung, and positive statistical results between expression level and poor differentiation, lymphovascular invasion. In addition to previously published papers, it is certain that miR-183 plays a potential oncogenic role in lung adenocarcinomas. It is reasonable to infer that miR-183 contributes tumorigenesis and tumor progression, especially lymphovascular infiltration, based on these findings.

This study showed that miR-183 expression was significantly higher in adenocarcinoma with *EGFR* exon 19 mutation than in those without this mutation. As far as we know, it is the first paper to identify that overexpression of miR-183 is significantly associated with *EGFR* exon 19 mutated lung adenocarcinoma. There are some published evidences to support this result. *PTEN* was originally identified as a tumor suppressor gene, and miRNAs were known to be involved in the EGFR/PTEN/AKT pathway [32, 33]. In detail, miR-183 knockdown of tumor cell lines caused deregulation of a miRNA network composed of *miR-183-EGR1-PTEN* in synovial sarcoma, rhabdomyosarcoma, and colon cancer [29], and the zinc finger transcription factor *EGR-1* (early growth response-1), known as a gene essential for growth, proliferation, or differentiation [34], is a direct regulator of *PTEN* [35]. We assume that miR-183 could play an oncogenic role in conjunction with *EGFR* amplification, *PTEN* loss, and AKT activation. We are cautiously optimistic that the development of miR-183 new target therapy might overcome TKI-resistant lung cancer.

Expression of miR-210 is linked to tumor proliferation, migration, and poor clinical outcome in breast cancer [36], and miR-210 is highly expressed in most solid tumors, including late stages of lung cancer [37]. In this study, the oncogenic role of miR-210 was presumed from higher expression in lung adenocarcinoma tissue than normal lung and positive statistical association between miR-210 expression and T stage, TNM stage, poor differentiation. Increased miR-210 expression showed a higher tendency toward pleural invasion and heavy smokers, *EGFR* mutation group than wild type group. Though not negligible, the correlations were insufficient to establish statistical significance. From all these considerations, we suggest miR-210 may participate in tumorigenesis and tumor progression.

miR-210 promotes glycolytic pathway in order to facilitate more rapid tumor growth [38]. Chen et al. suggested that cells with high miR-210 expression were significantly responsible to agents that inhibit glycolytic pathway, *in vitro* [39]. It is reasonable to assume that pulmonary adenocarcinomas with high miR-210 expression, which were found to be related to aggressive behavior in this study, will surely be a target that can be treated with anti-glycolytic agents.

In this study, we acknowledge that samples have limitations in the number, especially limited nodal or distant metastatic cases because of surgically resectable tumor, tumor histology (adenocarcinoma), and follow-up duration. More detailed studies on miRNAs associated with resistance of TKI treatment in *EGFR* mutated NSCLC could be needed in the future, miRNAs may be a breakthrough beyond the limitations of TKI treatment. In addition, new approach such as next-generation sequencing may provide us tools for better treatment protocols.

## Conclusions

In conclusion, we show that miR-34c may act as a potential tumor suppressor gene and miR-183 and miR-210 have a potential oncogenic role, using FFPE samples with various histopathologic parameters. This study also suggests different miRNA expression between *EGFR* mutation group and wild type group. Consequently, further studies of the biology of miRNAs may lead to diagnostic and prognostic biomarkers in pulmonary adenocarcinoma.

### Consent

Written informed consent was obtained from the patient for publication of this paper and any accompanying images. A copy of the written consent is available for review by the Editor-in Chief of this journal.

## Abbreviations

NSCLC: Non-small cell lung cancer
miRNA: microRNA
*EGFR*: Epidermal growth factor receptor
TKI: Tyrosine kinase inhibitor
FFPE: Formalin-fixed paraffin-embedded
TTF-1: Thyroid transcription factor
CDK: Cyclin-dependent kinase
Bcl-2: B-cell lymphoma 2
Bmf: Bcl-2-modifying factor
*EGR1*: Early growth response protein 1
*PTEN*: Phosphatase and tensin homolog
